# Clinical analysis and literature review of two paediatric cases of anti-IgLON5 antibody-related encephalitis

**DOI:** 10.3389/fneur.2024.1388970

**Published:** 2024-05-03

**Authors:** Mei Feng, Zhen Zhou, Qingyun Kang, Miao Wang, Jingwen Tang, Liwen Wu

**Affiliations:** Department of Neurology, The Affiliated Children’s Hospital of Xiangya School of Medicine, Central South University (Hunan children’s hospital), Changsha, China

**Keywords:** children, anti-IgLON5 antibody, autoimmune encephalitis, sleep disturbances, immunotherapy

## Abstract

**Introduction:**

Anti-IgLON5 antibody-related encephalitis is a rare autoimmune disorder of the central nervous system, predominantly occurring in middle-aged elderly individuals, with paediatric cases being exceptionally rare. This study aims to enhance the understanding of paediatric anti-IgLON5 antibody-related encephalitis by summarising its clinical and therapeutic characteristics.

**Method:**

A retrospective analysis was conducted on two paediatric patients diagnosed with anti-IgLON5 antibody-related encephalitis at Hunan Children’s Hospital from August 2022 to November 2023. This involved reviewing their medical records and follow-up data, in addition to a literature review.

**Results:**

The study involved two patients, one male and one female, aged between 2.5 and 9.6 years, both presenting with an acute/subacute course of illness. Clinically, both exhibited movement disorders (including dystonia, involuntary movements, and ataxia), cognitive impairments, sleep disturbances, and psychiatric symptoms. Patient 1 experienced epileptic seizures, while Patient 2 exhibited brainstem symptoms and abnormal eye movements. Neither patient showed autonomic dysfunction. Patient 1 had normal cerebrospinal fluid (CSF) and Brain MRI findings, whereas Patient 2 showed moderate leukocytosis and mild protein elevation in the CSF, and Brain MRI revealed symmetrical lesions in the basal ganglia and cerebellum. Oligoclonal bands in the CSF were positive in both cases. Both patients tested negative for HLA-DQB*05:01 and HLA-DRB*10:01. They received both first-line and second-line immunotherapies, with Patient 2 showing a poor response to treatment.

**Discussion:**

Paediatric cases of anti-IgLON5 antibody-related encephalitis similarly present sleep disturbances as a core symptom, alongside various forms of movement disorders. Immunotherapy is partially effective. Compared to adult patients, these paediatric cases tend to exhibit more pronounced psychiatric symptoms, a more rapid onset, and more evident inflammatory changes in the CSF. The condition appears to have a limited association with HLA-DQB*05:01 and HLA-DRB*10:01 polymorphisms.

## 1 Introduction

Anti-IgLON5 antibody-related encephalitis, also known as anti-IgLON5 syndrome, is a rare autoimmune disorder of the central nervous system with sleep disturbances as its core symptom. This condition was first reported in 2014 by Sabater et al. (1) in eight patients, primarily characterized by sleep disorders. It falls under the category of autoimmune encephalitis associated with anti-neuronal surface proteins. Unlike other conditions mediated by antibodies against neuronal surface receptors, which lead to reversible receptor reduction, the binding of anti-IgLON5 antibodies to IgLON5 results in an irreversible reduction of IgLON5 (2). Consequently, anti-IgLON5 antibody-related encephalitis is also considered to be linked with neurodegeneration. Almost all reported cases have been in middle-aged and elderly patients, with paediatric cases being exceedingly rare. This article presents an analysis and summary of the clinical data of two paediatric patients with anti-IgLON5 antibody-related encephalitis.

## 2 Materials and methods

The clinical data of two paediatric patients diagnosed with anti-IgLON5 antibody-related encephalitis in the Department of Neurology, Hunan Children’s Hospital from August 2022 to November 2023 were retrospectively analyzed. Both patients met the diagnostic criteria for autoimmune encephalitis in the paediatric patient (3).

## 3 Results

### 3.1 Clinical presentation

The study involved two paediatric patients, one male and one female, aged 9 years and 7 months, and 2 years and 6 months, respectively. Their conditions had durations of half a month and six days, respectively. Both patients had preceding infections. Patient 1 initially showed cognitive impairment, while Patient 2 presented with ataxia.

Patient 1, a 9-year-old girl, was admitted to the hospital on October 27, 2023, due to fever, memory deterioration over two weeks, convulsions, and behavioral abnormalities over a week. She experienced recurrent fever for two days two weeks prior, followed by memory decline, slowed thinking, forgetfulness, and difficulty concentrating after her temperature normalized. One week before admission, she had her first seizure, followed by progressive memory loss, involuntary movements of the right limb, frequent salivation, incoherent speech, and sleep difficulties (only 2 h per day). On admission, she was conscious but lethargic, intermittently responsive to simple questions but unable to follow commands. Her judgment and calculation were impaired, although muscle strength and tone were normal, occasionally experiencing claudication of the right lower extremity. Post-admission, she exhibited pronounced psychiatric symptoms including paroxysmal mania, mood swings, aggression, and loss of recognition of relatives. Subsequently, she lost the ability to communicate verbally or through eye contact and lost control of basic bodily functions. In this deteriorating state, she experienced involuntary movements, paroxysmal dystonia of the right lower extremity, and another convulsion.

Patient 2, a 2-year-old boy, was admitted to the hospital on August 15, 2022, due to a six-day fever and three-day gait disturbance. He experienced recurrent high fever and vomiting six days before admission, followed by the onset of gait disturbance. On admission, he appeared lethargic and mentally impaired, unable to sit or stand unaided. His muscle strength was graded at 4 in the upper limbs and 3 in the lower limbs, indicating reduced muscle tone, weakened tendon reflexes, and positive neck resistance. Following admission, the patient’s muscle strength progressively declined, reaching a nadir of 2 in the upper limbs and 0 in the lower limbs, with significant muscle hypotonia. He developed deep, hoarse voice and swallowing difficulties, eventually becoming mute. Additionally, he experienced hypermyotonia and involuntary movements over time. Throughout the course, he exhibited other symptoms including paroxysmal involuntary eye movements and tremors, loss of active language and communication, daytime agitation and irritability, nocturnal crying and restlessness, with frequent awakenings impairing sleep.

Both paediatric patients demonstrated normal neurodevelopment and motor skills, with no significant past medical history. Table 1 outlines the clinical characteristics of the two patients with anti-IgLON5 Antibody-Related Encephalitis.

**Table 1:** Clinical characteristics of two paediatric patients with anti-IgLON5 antibody-related encephalitis.

Case	Psychiatric disorders	Sleep disorders	Brainstem symptoms	Motor disturbances	Cognitive impairments	Abnormal eye movements	Convulsive seizures	Autonomic nervous system dysfunction
1	+	+	−	+	+	−	+	−
2	+	+	+	+	+	+	−	−

### 3.2 Auxiliary examinations

The two patients underwent various auxiliary examinations, including cerebrospinal fluid analysis, serum and cerebrospinal fluid anti-IgLON5 antibody titers, brain MRI, electroencephalogram, and HLA genotyping. Additional tests for autoimmune encephalitis-related antibodies and central demyelinating antibodies, such as anti-NMDAR, anti-CASPR2, anti-LGI1, anti-AMPA, anti-GABAB, anti-GABAA, anti-DPPX, anti-mGluR1, anti-mGluR5, anti-GAD65, anti-MOG, anti-GFAP, and anti-AQP4 antibodies, yielded negative results in both patients. Thoracic, abdominal, and pelvic CT scans revealed no evidence of tumors. Patient 2’s WES did not identify any pathogenic variations. Table 2 presents the details of the investigations conducted on the two patients.

**Table 2:** Investigations, treatment and response of the 2 patients.

Case	CSF findings (WBC; protein)	Titer of anti-IgLON antibody (serum/csf)	Brain MRI findings	VEEG findings	Gene	Treatment	Max/terminal mRs scores
1	6*10^6; 170 mg/L	1:100/1:10	Normal	Slow waves and epileptiform discharges	DQB1*05:01− DRB1*10:01− DQB1*03:01+ DRB1*15:01+	IVIG+IVMP+RTX	3/1
2	300*10^6; 690 mg/L	1:30/negative	T2/FLAIR hyperintense signals in the bilateral cerebellar hemispheres, basal ganglia, and dorsal thalamus	Slow EEG background	DQB1*05:01− DRB1*10:01− DQB1*05:02+ DRB1*16:01+	IVIG+IVMP+RTX + MMF	5/4

### 3.3 Immunotherapy and follow-up

**Figure 1:**
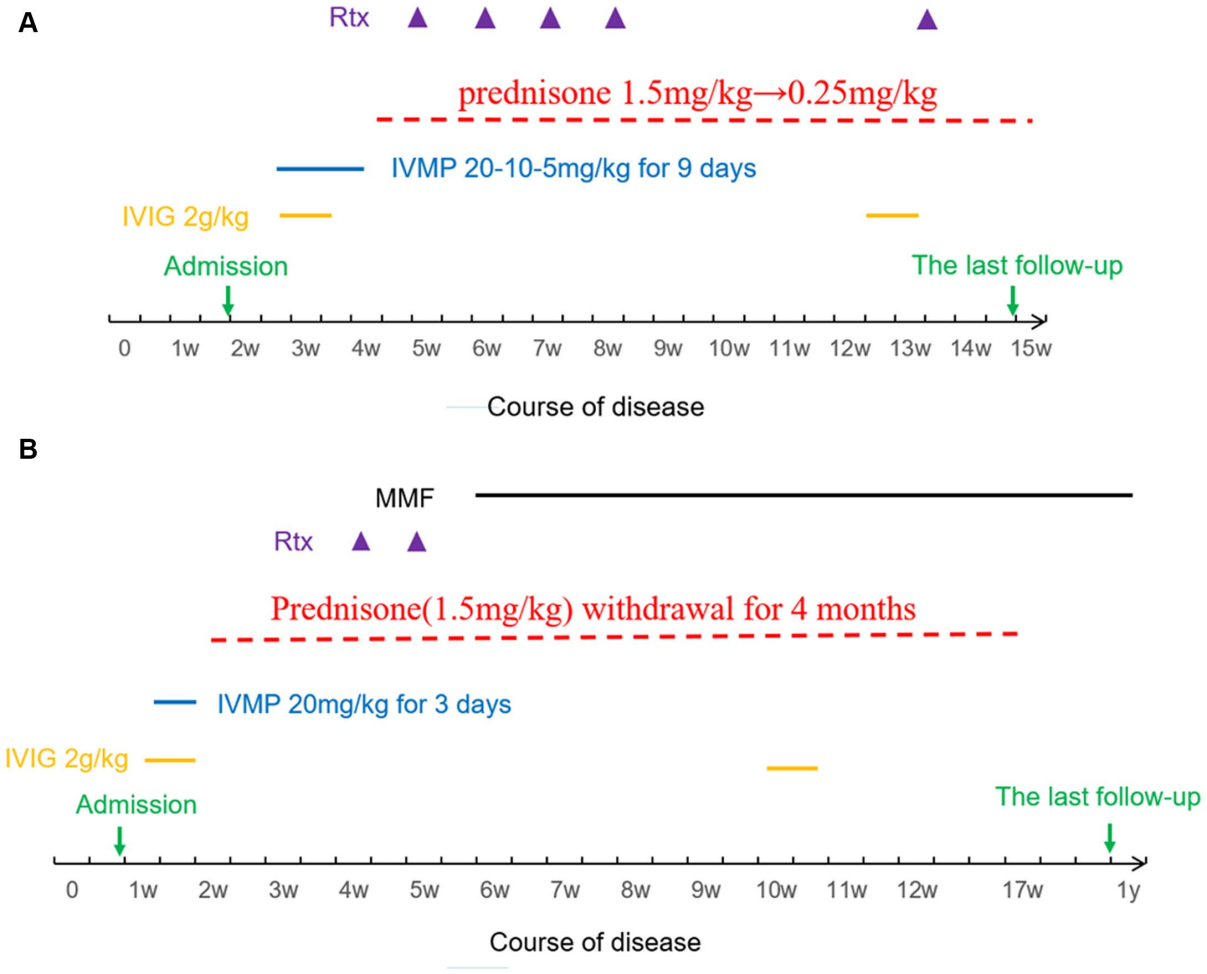
**(A)** Immunotherapy sequence for Patient 1. **(B)** Immunotherapy sequence for Patient 2. IVIG, Intravenous Immunoglobulin; IVMP, Intravenous Methylprednisolone; Rtx, Rituximab; MMF, Mycophenolate Mofetil.

Both patients underwent combination therapy comprising first-line and second-line immunotherapy. Figure 1A illustrates the details of Patient 1’s immunotherapy, while Figure 1B outlines Patient 2’s treatment regimen.

**Figure 2:**
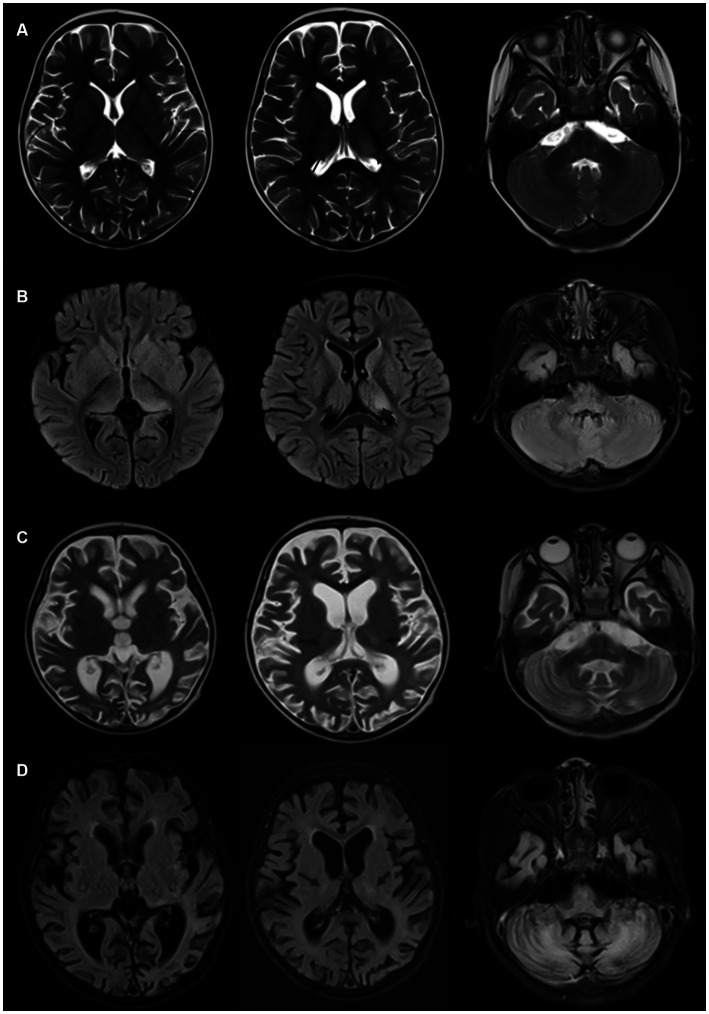
**(A,B)** Brain MRI of Patient 2, acquired one week after onset, demonstrating T2 and FLAIR hyperintense signals in the basal ganglia, dorsal thalamus, and bilateral cerebellar hemispheres. **(C,D)** Brain MRI of Patient 2, obtained one year after onset, showing a reduction in the original lesions in the basal ganglia and dorsal thalamus, along with marked global cerebral atrophy and significant ventricular dilation.

The modified Rankin Scale (mRS) was utilized to assess neurological disability at the peak of illness (maximum score) and during the final follow-up (terminal score). Patient 1 was followed up for three months, whereas Patient 2 was followed up for one year. Table 2 presents the treatment details and maximum/terminal mRS scores for both patients (see Figure 2).

## 4 Discussion

IgLON belongs to the immunoglobulin family of cell adhesion molecules, with members of the IgLON family possessing three immunoglobulin-like domains and a glycosylphosphatidylinositol (GPI) anchor (4). IgLON5 is primarily expressed in the nervous system and is located on human chromosome 19 (5). Its function remains unclear, but according to Sabater et al. (1), it may be associated with the physiology of Tau protein. Sabater et al. (2) found that the binding of anti-IgLON5 antibodies to IgLON5 on the neuronal surface can lead to an irreversible reduction of IgLON5, which is distinct from the reversible receptor reduction mediated by other neuronal surface receptor antibodies. Animal studies have also demonstrated (6) that anti-IgLON5 antibodies can disrupt the cytoskeletal structure of cultured rat hippocampal neurons, leading to neuritic dystrophy and axonal swelling. The role of IgLON cell adhesion molecules in neurodegenerative diseases has been confirmed (7). You et al. (8) found that direct injection of anti-IgLON5 antibodies into mice induces long-term neuronal damage and degenerative changes, as well as provokes an inflammatory response in the brain.

Tauopathies are a heterogeneous group of neurodegenerative disorders, pathologically characterized by neuronal and/or glial inclusions of the microtubule-binding protein, tau. They have been classified into three groups, including primary tauopathies, secondary tauopathies and geographically isolated tauopathies (9). Tauopathies are a heterogeneous group of neurodegenerative disorders, pathologically characterized by neuronal and/or glial inclusions of the microtubule-binding protein, tau. They have been classified into three groups, including primary tauopathies, secondary tauopathies and geographically isolated tauopathies (10). Anti-IgLON5 antibody-related encephalitis is a secondary tauopathy stands at a critical juncture of autoimmunity and neurodegeneration, where deposition of hyperphosphorylated tau occurs mainly in the hypothalamus, brainstem, and hippocampus (11). This suggests a potential link between autoimmunity and neurodegeneration in anti-IgLON5 disease, offering a new direction for research into the interrelationship between antibody-mediated neuroimmunity and neurodegeneration.

anti-IgLON5 antibody-related encephalitis predominantly affects middle-aged and elderly individuals aged 50–60 years, with no significant gender disparity observed. The disease course is generally chronic, though a minority of cases may present with acute or subacute onset. There have been very few reports of paediatric cases. Grüter et al. (12) summarised 53 cases of anti-IgLON5 antibody-related encephalitis, with an average age of 63.8 ± 10.3 years (ranging from 40–82 years). The duration from onset to diagnosis was 33.3 ± 37.5 months, and 15 out of 53 patients had a disease course within 4 weeks. Gaig et al. (13) reviewed 72 patients, noting an onset age of 62 years (ranging from 42–91 years), with only 3 out of 72 patients experiencing onset before the age of 50. The average time from onset to diagnosis was 36 months (ranging from 1–216 months), and 55 out of 72 patients had a disease course exceeding 4 weeks. In 2021, Ye et al. (14) reported a case of a 2-year-old child, which is the youngest reported case of anti-IgLON5 antibody-related encephalitis to date. This patient, diagnosed with Langerhans Cell Histiocytosis (LCH), developed neurological symptoms during the disease and was diagnosed with anti-IgLON5 antibody-related encephalitis following the detection of positive anti-IgLON5 antibodies at 1: 30 in the serum. The two paediatric patients in this study, one male and one female, included a case where symptoms began at 2.5 years of age, with a rapid onset and peak disease progression within approximately one week. The paediatric case reported by Ye et al. (14) was diagnosed over two months post-onset.

Although the pathogenesis of anti-IgLON5 antibody-related encephalitis differs from other types of autoimmune encephalitis and tends to have a longer disease course, it is still associated with viral infections. Li et al. (15) reported a case of anti-IgLON5 antibody-related encephalitis following novel coronavirus infection. Wang et al. (16) detected anti-IgLON5 antibodies in the cerebrospinal fluid of a patient with herpesvirus encephalitis. Both domestic (15) and international (17) reports have documented cases of anti-IgLON5 antibody overlap syndrome with LGI1 antibodies. Chung et al. (18) reported a case of anti-IgLON5 antibody-related encephalitis with positive GABA-B antibodies in the serum. The two patients in this study developed the condition following infection, and tests for NMDAR, CASPR2, LGI1, AMPA, GABA, DPPX, mGluR, GAD65, MOG, GFAP, and AQP4 antibodies were all negative.

The clinical presentation of anti-IgLON5 antibody-related encephalitis is diverse (19), with sleep disturbances as the hallmark symptom. Most patients exhibit various forms of movement disorders, including Parkinsonian symptoms, chorea, dystonia, involuntary movements, tremors, myoclonus, and ataxia. Commonly observed are dysarthria, dysphagia, vocal cord paralysis, and central hypoventilation, along with brainstem symptoms. Concurrently, patients may experience abnormal eye movements, cognitive dysfunction, and autonomic nervous system dysfunction. In the study by Gaig et al. (19), of 22 patients presenting to neurology, 8 had unstable gait as the primary symptom, and only 1 had cognitive decline. Paediatric cases display similar symptoms; the case reported by Ye et al. (14) in a child included predominant features of sleep disturbances, reduced speech, cognitive impairment, ataxia, and nystagmus. In this article, Patient 1 presented with cognitive dysfunction and epileptic seizures, while Patient 2’s initial symptom was ataxia. Both patients subsequently developed sleep disturbances, psychiatric symptoms, cognitive impairments and movement disorders, including dystonia, involuntary movements, and ataxia (only Patient 2), but without autonomic dysfunction. Patient 1 experienced two epileptic seizures during the course of the disease but did not require antiepileptic medication. Epileptic seizures are relatively rare in this condition; Wang et al. (20) noted that only 9 out of 183 patients had generalized/focal epileptic seizures, and in 6 patients, seizures were the primary reason for neurology consultation. Cases primarily presenting with intractable epilepsy have been reported (21). Both patients in this article had psychiatric disorders, while only 9 out of the 53 patients surveyed by Grüter et al. (12) exhibited psychiatric symptoms, suggesting that psychiatric manifestations might be more prominent in paediatric patients.

Most patients with this condition have normal or nonspecific changes in cranial imaging. Gaig et al. (19) reported that 18 out of 22 patients had normal or nonspecific changes in Brain MRI, with others showing mild brainstem or bilateral hippocampal atrophy. Carles et al. (13) drew a similar conclusion, with 58 out of 70 patients showing normal or nonspecific changes in Brain MRI, 6 with brainstem atrophy, and 3 with cerebellar atrophy. The paediatric case reported by Ye et al. (14) showed meningeal enhancement on Brain MRI but no cerebral parenchymal lesions. In the two cases discussed in this article, Patient 1 had normal cranial imaging, while Patient 2 showed significant changes, with symmetrical abnormal signals in the bilateral cerebellar hemispheres, basal ganglia, and dorsal thalamus, and notable cerebral atrophy post-treatment. Cases with significant Brain MRI abnormalities have been reported, such as the case by Han et al. (21) showing symmetrical abnormal signals in the dorsal cerebrum and deep white matter of the cerebellum, including the upper part of the cerebellum and ventrolateral thalamus. Patient 2, who developed symptoms post-infection, had a young age of onset, acute onset, rapid progression, and symmetrical intracranial lesions. Despite thorough metabolic assessments and whole-exome sequencing, no evidence of a metabolic encephalopathy was found. The significant cerebral parenchymal changes observed might be associated with the poor response to immunotherapy and unfavorable prognosis.

Sleep disturbances are a cardinal symptom of anti-IgLON5 antibody-related encephalitis (19). Sabater et al. (1) observed that abnormal sleep behaviors are the most common sleep disturbances in this condition, including sleep apnea, movement and complex behaviors, and snoring (22, 23), which may even necessitate ventilatory support. In this article, both patients exhibited varying degrees of sleep disturbances, such as reduced sleep and difficulty initiating sleep, treated with clonazepam and melatonin to improve sleep quality. Post-immunotherapy, both patients continued to experience sleep issues. Neither patient underwent comprehensive polysomnography (PSG) testing, but normal breathing and oxygen saturation during sleep suggested the absence of significant hypoxemia or sleep apnea.

According to Gaig et al. (19), 30% of patients exhibit elevated cerebrospinal fluid (CSF) white blood cell counts (>5 × 10^6^/L), 50% show mild increases in CSF protein, and 7% present oligoclonal bands in the CSF. Similar findings were reported by Grüter et al. (12), with 9 out of 51 patients showing CSF inflammatory changes, the highest white cell count being 56, oligoclonal bands positive in 6 out of 47 patients, and 24 out of 51 with elevated CSF protein. In this article, Patient 2 had a CSF white cell count of 300*10^6/L and mild protein elevation, with both patients showing positive oligoclonal bands, indicative of intrathecal synthesis. This suggests that paediatric patients, due to the acute onset of the disease, may exhibit more pronounced inflammatory changes in the CSF. Grüter et al. (12) found that the Cell-Based Assay (CBA) method detected anti-IgLON5 antibodies in the serum of 52 out of 53 patients, but only in the CSF of 5 out of 44 patients. The paediatric case reported by Ye et al. (14) only showed positive serum antibodies, which might be related to pre-test steroid use. In this article, both paediatric patients had positive serum antibodies, with only one patient showing positive CSF antibodies, aligning with the findings in the literature.

Anti-IgLON5 antibody-related encephalitis is an autoimmune disease. In a study by Gaig et al. (13), 6 out of 72 patients had concurrent other autoimmune diseases. This condition can coexist with malignancies in various locations, though the incidence rates reported in the literature vary. Grüter et al. (12) found that 8 out of 53 patients had different types of malignancies, with 4 cases occurring before the onset of anti-IgLON5 disease and 4 diagnosed post-encephalitis diagnosis. The paediatric case reported by Ye et al. (14) was diagnosed during chemotherapy for LCH. In this article, both paediatric patients were tested for common autoimmune disease antibodies, all of which were negative, and thoracic, abdominal, and pelvic CT scans did not reveal any systemic tumors. This could suggest that, similar to other types of autoimmune encephalitis, the incidence of concurrent tumors in paediatric patients might be relatively lower, which warrants further verification through more paediatric case studies and extended follow-up.

In 2014, Sabater et al. (1) performed genetic testing on 4 patients with anti-IgLON5 antibody-related encephalitis. All 4 patients were positive for the HLA-DQB*05:01 and HLA-DRB*10:01 alleles, suggesting that genetic predisposition may play a significant role in the pathogenesis of this disease. Gaig et al. (19) conducted genetic tests on 15 patients, with 13 out of 15 being positive for HLA-DRB*10:01 and HLA-DQB*05:01. The frequency of the HLA-DRB*10:01 allele was 36 times higher than in the general population, and HLA-DQB*05:01 was 3.5 times higher. In Gaig et al.’s (13) report, 36 out of 62 patients were positive for the HLA-DRB*10:01 allele, and 61 out of 62 detected the HLA-DQB*05:01 allele. The paediatric case reported by Ye et al. (14) was negative for both HLA-DQB*05:01 and HLA-DRB*10:01. The two paediatric patients in this article were also negative for HLA-DQB*05:01 and HLA-DRB*10:01 alleles, suggesting that the correlation with HLA-DQB*05:01 and HLA-DRB*10:01 polymorphisms might be less significant in paediatric patients compared to adults. However, due to the limited number of paediatric cases, further research with more cases is required. Additionally, Gaig et al. (24) found that patients carrying the HLA-DRB*10:01 allele were more likely to exhibit sleep abnormalities, medullary dysfunction, and autonomic nervous system disorders, while cognitive impairment was more associated with the absence of the HLA-DRB*10:01 allele. Both patients in this article had significant cognitive dysfunction, which might be somewhat correlated with the negative HLA-DRB*10:01 status. Despite the negativity of the two classical subtypes in these two cases, Patient 1 was positive for HLA-DQB*03:01 and DRB*15:01, and Patient 2 for HLA-DQB*05:02 and DRB*16:01. The HLA-DQB*03:01 allele is associated with a 1.5-fold (25) increased risk of Narcolepsy Type 1 (NT1) and an earlier onset of NT1 by 2 years (26). The HLA-DRB*15:01 allele is also considered a risk factor for NT1 (27), and immunosuppressive treatment can induce narcolepsy in genetically susceptible patients (28). The HLA-DQB*05:02 allele is highly associated with AQP4-positive Neuromyelitis Optica Spectrum Disorder (NMOSD) in the Chinese population (29) and is a high-risk factor for the onset of Myasthenia Gravis (30), particularly in MuSK-related Myasthenia Gravis (31).

Anti-IgLON5 antibody-related encephalitis is known for its poor response to immunotherapy, unfavorable prognosis, and high mortality rate. The most common causes of death include central hypoventilation, unexplained sudden death during sleep or wakefulness, bradycardia, and aspiration pneumonia resulting from dysphagia (32). Immunotherapy encompasses first-line and second-line treatments, and it is imperative to commence immunotherapy promptly upon diagnosis. Data from one study (33) indicate that 20 out of 46 patients responded to immunotherapy, with the most common regimens including corticosteroids, IVIG, plasma exchange, rituximab, cyclophosphamide, azathioprine, and mycophenolate mofetil. Given that the disease is mediated by the Immunoglobulin G4 (IgG4) subclass, which does not activate complement or complement-mediated immune responses, monotherapy with IVIG often results in suboptimal improvement. However, Logmin et al. (34) reported a patient with predominant cognitive impairment and chorea who showed substantial clinical recovery to normal levels after early treatment with high-dose methylprednisolone, followed by monthly IVIG maintenance for 6 months. Studies (35) suggest that combination and second-line therapies appear more effective than monotherapy. Madetko et al. (36) recommend a combination of IVIg, plasma exchange, and rituximab as the preferred treatment regimen. A case reported in the New England Journal of Medicine in 2022 (37) showed improvement in sleep disturbances but not in ataxia and cognitive impairment, following acute-phase treatment with steroids and IVIG, followed by maintenance therapy with rituximab. Data from Grüter et al. (12) indicate that 41% of patients with acute exacerbations of a chronic course showed clinical improvement post-immunotherapy, especially when treated within 6 weeks. In patients who commenced long-term immunotherapy, 75% did not exhibit further progression of symptoms. Single and multivariate regression analyses confirmed that the effectiveness of immunotherapy might be associated with factors such as a subacute disease course, low pre-treatment NFL levels, and initiation of long-term immunotherapy within one year of onset. Madetko et al. (36) suggest that factors potentially enhancing the effectiveness of immunotherapy include cognitive impairment, non-classical phenotypes, HLA-DQB *05:01 (+) and HLA-DRB *10:01 (−) status, IgG1 subtype, and inflammatory changes in the cerebrospinal fluid. In this article, both paediatric patients presented with acute/subacute disease courses, received timely immunotherapy, and initiated second-line treatment early. Despite the apparent inflammatory changes in the CSF of Patient 2, the response to immunotherapy was poor, and recovery was limited. Following extensive rehabilitation over more than a year, Patient 2 achieved only a mRS score of 4, possibly due to multiple intracranial lesions and severe brain damage. As the analysis of immunotherapy effectiveness in the literature primarily pertains to middle-aged and elderly individuals, the effectiveness and influencing factors of immunotherapy in paediatric patients may differ, necessitating further research with larger sample sizes for validation and confirmation.

In summary, anti-IgLON5 antibody-related encephalitis is a rare and relatively unique form of autoimmune encephalitis. It is characterized by irreversible neurodegeneration, which contributes to the suboptimal response to immunotherapy. The disease predominantly affects middle-aged and elderly individuals, with paediatric cases being exceedingly rare. In children, anti-IgLON5 antibody-related encephalitis primarily presents with sleep disturbances and various forms of movement disorders, and there is partial effectiveness of immunotherapy. Compared to adult patients, paediatric cases tend to exhibit more pronounced psychiatric symptoms, a more acute onset of the disease, and more evident inflammatory changes in the cerebrospinal fluid. There appears to be a lesser association with HLA-DQB *05:01 and HLA-DRB *10:01 polymorphisms in children. Further research and a larger number of cases are needed to substantiate these observations.

## Data availability statement

The datasets presented in this study can be found in online repositories. The names of the repository/repositories and accession number(s) can be found in the article/supplementary material.

## Ethics statement

The studies involving humans were approved by Medical Ethics Committee of Hunan Children’s Hospital. The studies were conducted in accordance with the local legislation and institutional requirements. Written informed consent for participation in this study was provided by the participants’ legal guardians/next of kin. Written informed consent was obtained from the minor(s)’ legal guardian/next of kin for the publication of any potentially identifiable images or data included in this article.

## Author contributions

MF: Writing – review & editing, Writing – original draft, Software, Methodology, Investigation, Formal analysis, Data curation. ZZ: Writing – original draft. QK: Writing – review & editing. MW: Writing – original draft. JT: Writing – review & editing. LW: Writing – review & editing.
